# Diagnostic value of 3D power Doppler ultrasound in the characterization of thyroid nodules

**DOI:** 10.3906/sag-1803-92

**Published:** 2019-06-18

**Authors:** Ayşegül CANSU, Emin AYAN, Sibel KUL, İlker EYÜBOĞLU, Şükrü OĞUZ, Sevdegül MUNGAN

**Affiliations:** 1 Department of Radiology, Faculty of Medicine, Karadeniz Technical University, Trabzon Turkey; 2 Department of Radiology, Faculty of Medicine, Acıbadem University, Kayseri Hospital, Kayseri Turkey; 3 Department of Pathology, Faculty of Medicine, Karadeniz Technical University, Trabzon Turkey

**Keywords:** Thyroid nodules, three-dimensional power Doppler ultrasound, vascular indices, VOCAL

## Abstract

**Background/aim:**

This study aimed to evaluate the diagnostic value of vascular indices obtained using 3D power Doppler ultrasound (3D PDUS) in differentiation of benign and malignant thyroid nodules.

**Materials and methods:**

Sixty-seven patients (56 female, 11 male, mean age 44.6) with 81 thyroid nodules exhibiting mixed (peripheral and central) vascularization patterns, with the largest diameter between 10 and 30 mm, were prospectively evaluated using 3D PDUS. Nodule volume, vascularization index (VI), flow index (FI), and vascularization flow index (VFI) were calculated using the Virtual Organ Computer-aided Analysis (VOCAL) software, and these indices were then compared with regard to the cytohistopathology-based diagnosis. The optimum cutoff values for the differentiation of benign and malignant nodules were identified, and diagnostic efficacy was calculated using receiver operating characteristic (ROC) analysis.

**Results:**

Fifty-six of the 81 nodules included in this study were diagnosed as benign and 25 as malignant. Vascular indices in malignant nodules were significantly higher than those in benign nodules (P < 0.05). In benign nodules, the mean VI was 11.61 ± 6.88, mean FI was 39.75 ± 3.93, and mean VFI was 4.82 ± 2.94, compared to 18.64 ± 12.81, 41.82 ± 4.43, and 8.17 ± 6.37, respectively, in malignant nodules. The area under the curves (AUCs) was calculated as 0.68, 0.61, and 0.67 for VI, FI, and VFI, respectively. At optimal cutoff values of 10.2 for VI, 40.8 for FI, and 5.5 for VFI, the sensitivity and specificity were 72%/55.4%, 68%/57.1%, and 68%/67.9%, respectively.

**Conclusion:**

3D PDUS can be useful in the characterization of thyroid nodules.

## 1. Introduction

Thyroid nodules are very common, and their prevalence increases with age. The prevalence of thyroid nodules ranges from 30% to 60% in autopsy series and from 19% to 68% with high-resolution ultrasound (US) [1,2]. Despite the high prevalence of nodular thyroid disease, thyroid cancer is not a common condition. A risk for malignancy is observed in 7%–15% of cases, depending on factors such as age, sex, radiation exposure, family history, and the levels of thyroid hormones [3–5]. Conventional ultrasound (US) is the first-step imaging technique in the radiological evaluation of thyroid nodules. However, there is no single 100% reliable sonographic criterion for differentiating benign and malignant nodules at gray scale examination. A combination of gray scale characteristics is required in order to increase sensitivity and specificity, and fine needle aspiration (FNA) is needed for the final diagnosis when suspected nodules are present. However, FNA is also subject to false positive or negative results, and material obtained at FNA may be nondiagnostic [6]. Additional and new imaging modalities are therefore needed for more accurate diagnosis. Color Doppler ultrasonography (CDUS), three-dimensional ultrasound (3D US), three-dimensional power Doppler ultrasound (3D PDUS), contrast-enhanced ultrasound (CEUS), and sonoelastography are imaging techniques used in addition to conventional US.

Along with the developments in sonoelastography and 3D sonographic techniques, several different quantitative measurement parameters have been used in daily practice. The detection of elasticity index by elastography has been proposed as a supplementary tool in the assessment of thyroid nodules [7]. On the other hand*,* the use of 3D US and 3D PDUS apart from obstetric radiology has become increasingly widespread in recent years. Three-dimensional US, 3D PDUS, and computer-aided color Doppler software methods provide a number of advantages over conventional techniques [8,9]. Three-dimensional US is more accurate than two-dimensional examination in the evaluation of anatomical structures and assessment of disease and provides data that can be subjected to repeated evaluation [8,9]. Three-dimensional power Doppler US is able to quantify vascularity within organs, tissues and, tumors [10,11]. From the data obtained with 3D PDUS, vascular indices can be calculated using the Virtual Organ Computer-aided Analysis (VOCAL) software, and tissue vascularization can be shown with numerical values using these indices. This software provides 3 automatically calculated vascular indices as vascularization index (VI), flow index (FI), and vascularization flow index (VFI) [12].

Numerous studies have investigated benign–malignant differentiation in gynecological and breast masses using 3D PDUS [11,13–16]. However, to the best of our knowledge, few studies have evaluated thyroid nodules using 3D US and 3D PDUS [17–20]. Of these, only one has assessed thyroid nodule vascularization in a quantitative manner [18]. The purpose of this study was to investigate the diagnostic value of vascular indices obtained using 3D PDUS in differentiating benign and malignant thyroid nodules.

## 2. Materials and methods

### 2.1. Subjects

The present study was approved by the local ethical committee and informed consent of the patients was obtained. One hundred and fourteen thyroid nodules with mixed (peripheral and central) vascularization patterns, with the largest diameter between 10 and 30 mm, in 90 patients referred for FNA were prospectively assessed using 3D PDUS. This criterion of largest diameter was intended to eliminate the volumetric discrepancy between benign and malignant nodules. On the other hand, nodules smaller than 10 mm could not be evaluated properly using power Doppler. Definite diagnosis (benign or malignant nodule) was made with FNA and/or histopathological analysis after surgery. Thirty-three nodules were excluded from the study due to retrosternal extension (n = 13), nondiagnostic material (n = 11), or respiratory or carotid pulsation artifacts (n = 9). The final study group included 81 nodules in 67 patients (56 female, 11 male; aged 22–61 years; mean age 44.6).

### 2.2. Ultrasound examination

**Figure 1:**
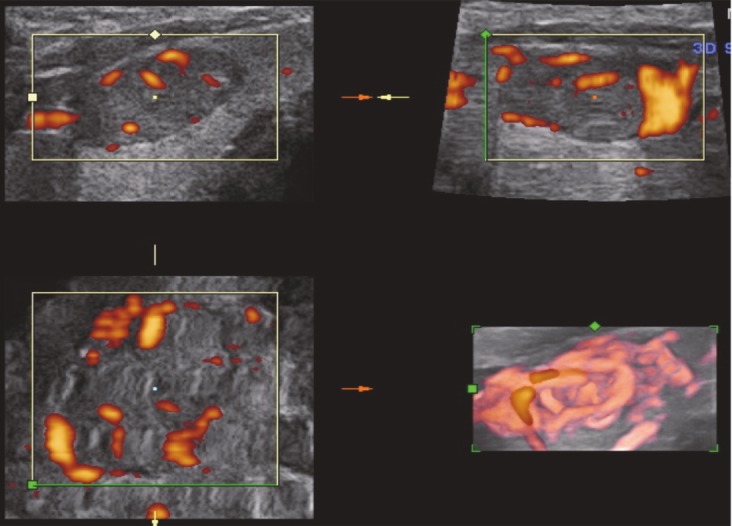
Sonogram showing 3D power Doppler acquisition in the axial plane using 3D power Doppler ultrasound. The power Doppler box was positioned so as to cover the entire thyroid nodule. The widest scanning angle (29°) including the whole thyroid nodule was used.

**Figure 2:**
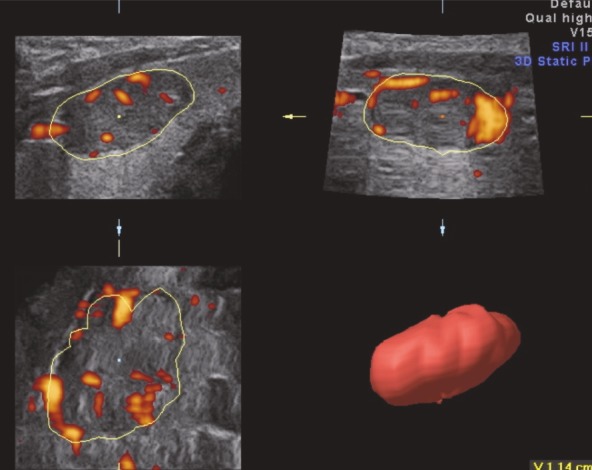
Using the manual contour method in the VOCAL technique, the outer margins of the nodule were drawn on a fixed axis with a 15° angle of rotation. Once all the contours had been traced, the volume of the nodule was calculated automatically (the volume of the thyroid nodule can be seen in the lower right-hand corner).

**Figure 3:**
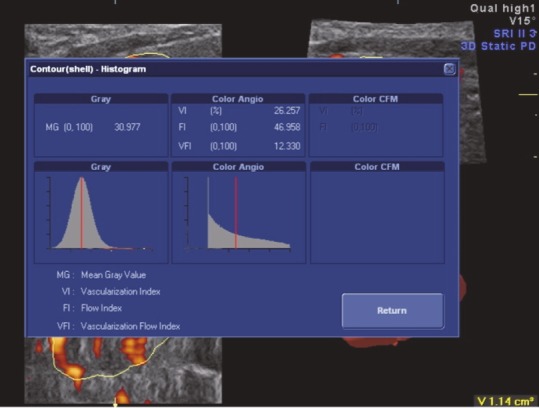
Three-dimensional power Doppler images were analyzed using the VOCAL histogram software. The results of the histogram analysis of the thyroid nodule show values for the vascularization index (VI), flow index (FI), and vascularization flow index (VFI).

The examinations were performed using a Voluson 730 Expert (General Electric, Waukesha, Wisconsin) device with a 6–12 MHz volumetric linear probe. In order to standardize our evaluation of nodule vascularization, the same power Doppler settings were used. After the visualization of the thyroid nodule in a 2D view, patients were requested to hold their breath for approximately 20 s, and 3D static power Doppler scanning was performed using the widest scanning angle (29°) covering the entire thyroid nodule (Figure 1). These acquired volume data were analyzed using the VOCAL software. The outer margins of the nodule were drawn manually on a fixed axis with a 15° angle of rotation by the same radiologist (Figure 2). When all contours were traced, the volume and three vascular indices (VI, FI, and VFI) of the thyroid nodule were calculated automatically using the histogram facility in the VOCAL software (Figure 3). VI represents the ratio of color voxels to all voxels and shows the density of vessels. FI is the sum of weighted color voxels divided by the number of all color voxels and reflects mean color intensity. VFI represents the sum of weighted color voxels divided by all voxels and represents vascularization and perfusion [12].

### 2.3. Statistical analysis

The means and ranges of nodule size, volume, and vascular indices of benign and malignant nodules were calculated. The vascular indices of benign nodules were compared with the vascular indices of malignant nodules using the independent samples test. The optimum cutoff values for the differentiation of benign and malignant nodules were determined using receiver operating characteristic (ROC) analysis. Sensitivity, specificity, positive predictive value (PPV), negative predictive value (NPV), and the area under the curve (AUC) for vascular indices were calculated with a 95% confidence interval. A P-value <0.05 was considered statistically significant. 

## 3. Results

Fifty-six (69%) of the nodules included in the study were diagnosed as benign and 25 (31%) as malignant. The analysis of the histopathology results revealed that all the malignant nodules were papillary carcinoma. Of the benign nodules, 19 cases were nodular colloidal goiter, 2 were lymphocytic thyroiditis, and 2 were Hurthle cell adenoma, the remaining cases being diagnosed as benign histopathology.

The mean age of the group with benign nodules was 46.4 **± **7.7 years, compared to 40.7 **± **13.2 in the group with malignant nodules. No statistically significant difference was determined between the two groups with regard to age (P = 0.53). The mean volume of benign nodules was 4.22 cm3, compared to the mean volume of 4.11 cm3 in malignant nodules in this study, among nodules with the largest diameter between 1 and 3 cm. The difference in mean volumes between the two groups was not statistically significant (P = 0.94). When the groups were evaluated in terms of nodule content, 32 of the benign nodules were solid (58%), 24 of them were solid predominant (42%), while 18 of the malignant nodules were solid (72%), 6 of them were solid predominant (24%), and 1 of them was cystic predominant (4%). Vascular indices were significantly higher in malignant nodules compared to benign nodules (P < 0.05). In benign nodules, the mean VI was 11.61 ± 6.88, the mean FI was 39.75 ± 3.93, and the mean VFI was 4.82 ± 2.94. In the malignant nodules, the mean VI was 18.64 ± 12.81, the mean FI was 41.82 ± 4.43, and the mean VFI was 8.17± 6.37 (Table 1).

**Table 1:** The comparison of nodule volume (NV) and vascular indices between benign and malignant thyroid nodules.

	Benign	Malignant	P-value
NV ( cm3)	4.2 ± 0.1	4.1 ± 0.9	0.94
VI	11.61 ± 6.88	18.64 ± 12.81	0.015
FI	39.75 ± 3.93	41.82 ± 4.43	0.039
VFI	4.82 ± 2.94	8.17 ± 6.37	0.018

Values are given as mean values ± SD; P: significance level for all pairs

**Figure 4:**
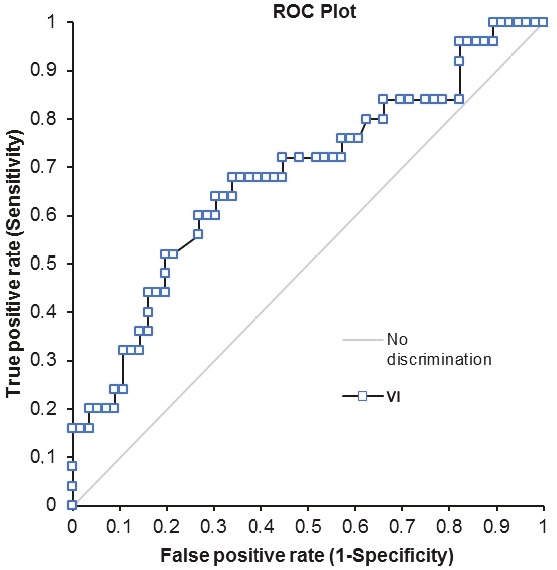
Graph showing the receiver operating characteristic curve for VI.

**Figure 5:**
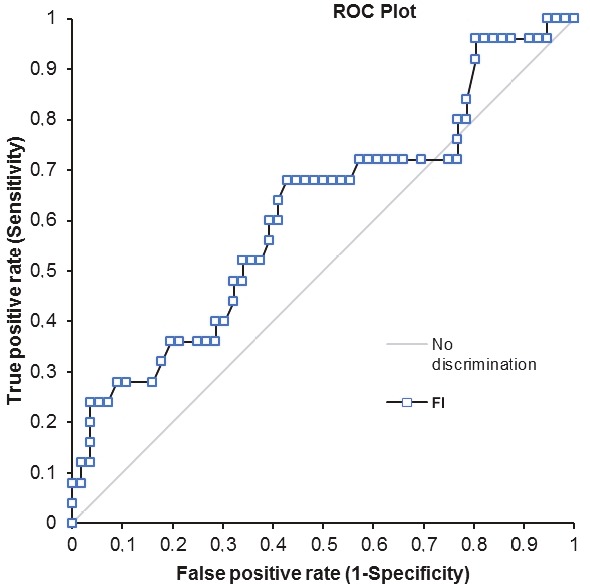
Graph showing the receiver operating characteristic curve for FI.

**Figure 6:**
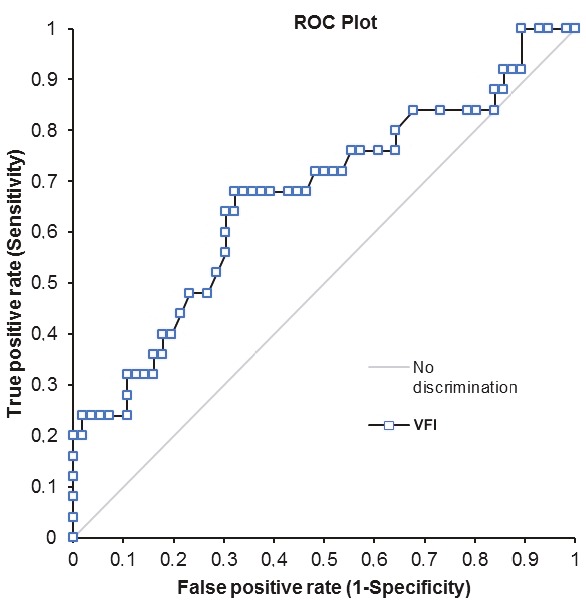
Graph showing the receiver operating characteristic curve for VFI.

The area under the ROC curve (AUC) values for VI, FI, and VFI were 0.68, 0.61, and 0.67, respectively. The ROC curves of the vascular indices are shown in Figures 4–6. At an optimal threshold value of 10.2 for VI, sensitivity was determined at 72% and specificity at 55.4%. At an optimal threshold value of 40.8 for FI, sensitivity was 68% and specificity 57.1%, while at an optimal threshold value of 5.5 for VFI, sensitivity was 68% and specificity 67.9%. The acquired diagnostic values for three vascular indices using the stated cutoff values are given in Table 2.

**Table 2:** Results of Receiver Operating Characteristic (ROC) Analysis for vascular indices.

	Cutoff level	Sensitivity (%)	Specificity (%)	PPV (%)	NPV (%)	AUC	95% CI
VI	10.2	72	55.4	41.9	81.6	0.68	0.415–0.687
FI	40.8	68	57.1	41.5	80	0.61	0.432–0.703
VFI	5.5	68	67.9	48.6	82.6	0.67	0.540–0.797

AUC: Area under curve; PPV: positive predictive value; NPV: negative predictive value; CI: confidence interval

## 4. Discussion

This study investigated the diagnostic value of 3D PDUS in differentiating between benign and malignant thyroid nodules. We analyzed the relations between nodule characterization and vascular indices (VI, FI and, VFI) obtained using the VOCAL software. A statistically significant difference was observed in terms of vascular indices between benign and malignant nodules, these indices being higher in malignant nodules. 

Two-dimensional color or power Doppler US has been used in the characterization of thyroid nodules in numerous studies, and central vascularization, which more commonly increases in malignant nodules, can be examined subjectively using these methods [7,21–23]. With the increasing use of 3DPDS in clinical research, mass vascularization can now be evaluated quantitatively, and significant differences are determined in the differentiation of benign and malignant lesions [11,13–16]. To the best of our knowledge, apart from the present research, Li et al. performed quantitative evaluation of benign and malignant nodules by using the VOCAL software [18]. In contrast to the findings of our study, they reported higher vascular indices in benign nodules. However, considering the tumoral vascularity of thyroid and nonthyroid masses, we think that these results are inconsistent [13–16,24–26]. In a study using a computer-aided color Doppler software, Baig et al. developed a new method that divided thyroid nodule into central and peripheral regions and computed the regional and entire vascular index (VI) of the nodule [9]. They found that the mean VI of malignant nodules was significantly higher than that of benign nodules, in agreement with our study [9]. On the other hand, studies involving the characterization of thyroid nodules with CEUS in which the microvascular flow pattern is evaluated in tumoral lesions support our own findings [24–26]. In a study of 46 patients, Nemec et al. observed a significant difference in contrasting between benign and malignant nodules and determined a sensitivity of 76.9%, specificity of 84.8%, and accuracy of 82.6% for CEUS based on ROC analysis [26]. The reconstruction and segmentation of CEUS imaging data indicated that malignant nodules have a higher internal vasculature [24] and a higher vascular density than benign nodules. Since the vascular indices obtained using 3D PDUS show the percentage of vascularized tissue and mean blood flow rate in tissue, we hypothesize that it can also be used for assessing tumoral angiogenesis with detected CEUS without using contrast material. In this study, which included suspected nodules with a mixed vascularization pattern with CDUS, although the vascularization patterns were subjectively similar, the fact that vascular indices of malignant nodules were relatively higher than those of benign nodules supports our hypothesis. Based on ROC analysis, the sensitivity of vascular indices was approximately 68%–72% and the specificity 55%–68%. 

Malignant thyroid nodules tend to have a rich intralesional vascularization with anarchic structure [25]. The vascular network in thyroid nodules can be visualized with 3D PDUS, although when the entire lesion is included in the examination, peripheral and central vascularization can overlap, particularly in intensely vascularized nodules. In order to eliminate this possibility, Slapa et al. identified risk factors for thyroid cancer using thin-slice volume rendering together with gray scale and 3D PDUS [17]. They analyzed the relationship between the central vascular density of thyroid nodules and malignity, and reported that central vascular density was 75%–81% sensitive and 49%–56% specific in identifying thyroid cancer. A combination of contrast-enhanced US and 3D US permits a more detailed evaluation of the vascular tree [24,26]. Molinari et al. developed an image processing technique for skeletonization of intranodular vascularization using 3D CEUS [25]. The parameters involved in the skeletons were the number of vascular trees, vascular density, the number of branching nodes, the mean vessel radius, tortuosity, and the inflection count metric. According to that study, malignant nodules had a higher number of vascular trees and branches and higher vascular density [26]. These results indicate that malignant lesions are perfused by a dense vascular bed, and this is in agreement with our results. Molinari et al. reported that the quantification of nodule vascularization with skeletonization using 3D CEUS may be a useful technique in the differential diagnosis of thyroid lesions [25]. However, to the best of our knowledge, no study has used this thin-slice reconstruction technique in comparing the effectiveness of noncontrast 3D PDUS and 3D CEUS. We think that for a more conclusive statement, further studies are needed to investigate this. Since the VOCAL software does not permit thin-slice rendering, vascular trees could not be evaluated in detail together with vascular indices.

The present study has some limitations. The first involves the known deficiencies of ultrasound, depending on the operator and patient. Artifacts deriving from respiratory movements and carotid pulsation prevented us from obtaining appropriate images in some patients. The second limitation is that the histopathological results from all malignancies indicated papillary carcinomas and the number of malignant lesions was lower than that of benign lesions. The third limitation is the exclusion of the nodules smaller than 1 cm or larger than 3 cm in diameter in order to reduce the volumetric effect on vascularization. The final limitation is that this technique is not appropriate for nodules with retrosternal extension.

In conclusion, vascular indices obtained using 3D PDUS provide useful and quantitative analysis for the differentiation of benign and malignant thyroid nodules without contrast agent administration. Further studies are needed to determine the contribution of 3D PDUS to conventional US and to compare the efficacy of CEUS and 3D PDUS in the characterization of thyroid nodules.
